# Matrix Metalloproteinase-9 as a Predictor of Healing in Diabetic Foot Ulcers

**DOI:** 10.7759/cureus.75521

**Published:** 2024-12-11

**Authors:** Prathvi Shetty, Rohan Dsouza, Vinoda Kumar B

**Affiliations:** 1 General Surgery, Father Muller Medical College, Mangalore, IND

**Keywords:** diabetic foot ulcers, glycosylated haemoglobin (hba1c), matrix metalloproteinase 9, matrix metalloproteinase 9 inhibitor, ­wound healing

## Abstract

Background

Wound healing in diabetic foot ulcers (DFUs) is hindered by several physiological and biochemical abnormalities, including prolonged inflammation, an imbalance in extracellular matrix (ECM) synthesis and degradation, insufficient neovascularization, and reduced macrophage activity. In DFUs, excessive and uncontrolled matrix metalloproteinases (MMPs) degrade the ECM and impede wound healing. Matrix metalloproteinase-9 (MMP-9) concentration plays a key role in inflammation and ECM degradation. This study explores the relationship between wound type in DFUs and MMP-9 levels, hypothesizing that a high MMP-9 environment may indicate inflammation and impaired wound healing.

Materials and methods

Forty individuals with type 2 diabetes and foot ulcers were recruited for the study. The participants were divided into two groups: nonhealing and healing, with 20 patients in each group. Biopsy samples were homogenized, and MMP-9 activity was measured using an ELISA.

Results

The MMP-9 concentration was significantly higher in nonhealing ulcers compared to healing ulcers. Receiver operating characteristic analysis revealed that MMP-9 measurement was the most accurate predictor of wound healing, with an area under the curve value of 0.945 and high sensitivity and specificity. Although there was a weak correlation between MMP-9 concentration and glycosylated hemoglobin, it was not statistically significant.

Conclusions

MMP-9 expression serves as a marker of poor wound healing. MMP-9 levels in the wound at the time of presentation may predict the healing trajectory and help tailor a specific treatment plan for each DFU patient.

## Introduction

One of the major long-term consequences of diabetes mellitus (DM) is diabetic foot ulcers (DFUs), which are also a significant cause of morbidity and mortality in developing nations [1]. Reduced blood flow to the lower limbs increases the risk of necrosis, infection, and deep tissue involvement in DFUs, all of which can lead to amputation [2]. DFU-related lower limb amputation rates are 10-20 times higher than those in nondiabetic individuals. The treatment of DFUs can be costly and time-consuming. Clinical research has shown that typical healing rates for DFUs with standard therapies range from 12% to 20% [3].

Wound healing in DFUs is hindered by several physiological and biochemical anomalies, including prolonged inflammation, imbalances in the synthesis and degradation of the extracellular matrix (ECM), inadequate neovascularization, and reduced macrophage activity [4-6]. Matrix metalloproteinases (MMPs) play a role in each of these processes [7]. MMPs are crucial for wound healing as they degrade the ECM; however, excessive and uncontrolled MMP activity in DFUs impedes healing by destroying the ECM [8-13]. Among these MMPs, MMP-9 is particularly important in inflammation and ECM degradation [14].

This study aims to explore the relationship between MMP-9 levels, wound type in DFUs, and hemoglobin A1C (HbA1c) levels, hypothesizing that a high MMP-9 environment may indicate inflammation and inadequate wound healing. Additionally, we are examining the correlation between MMP-9 levels and glycosylated hemoglobin (HbA1c) in DFU patients.

## Materials and methods

The study involved the recruitment of 40 individuals diagnosed with type 2 DM and foot ulcers. Each group consisted of 20 patients: one group with infectious DFUs and the other with healing DFUs. Microbiological tests and clinical presentation were used to identify the infectious state of the DFUs.

DFUs were classified as full-thickness skin lesions according to the International Working Group on the Diabetic Foot [15]. The severity of DFUs was graded using Wagner’s classification system: Grade 0, intact skin with a high risk of ulceration; Grade 1, superficial ulcer without clinical infection; Grade 2, deeper ulcer involving tendon, bone, or joint; Grade 3, typically involving bone tissue with abscess formation, osteomyelitis, or tendinitis; Grade 4, localized gangrene with ischemic ulcers; and Grade 5, extensive gangrene [16].

All patients provided informed consent to participate in the trial, which was approved by the Ethics Committee. Exclusion criteria included conditions that could interfere with MMP levels, such as lower limb arteriopathy and infection. Arteriopathy was identified by the absence of posterior tibial and pedal pulses or an ankle/brachial index of <0.9.

Each patient had a wound measuring at least 2 cm by 2 cm. A 5-mm punch biopsy was taken from the foot ulcer, and the samples were homogenized. MMP-9 activity was measured using an ELISA, and parameters such as sensitivity, specificity, accuracy, and consistency were analyzed.

## Results

A cross-sectional study was conducted involving 40 patients. No statistically significant differences were found in age, gender, HbA1c levels, or initial ulcer size between the healed and non-healed groups. However, the concentration of MMP-9 in the wound was significantly higher in nonhealing ulcers compared to healing ulcers (p = 0.000) (Table 1).

**Table 1:** Descriptive statistics for demographic characteristics, glycated hemoglobin, and MMP-9 levels based on wound healing status The p-value represents the significance level of the Mann-Whitney test for comparing the distribution between groups. Values are expressed as mean (SD). HbA1C: glycated hemoglobin; MMP-9: matrix metalloproteinase-9

Parameter	Healing (N = 20)	Nonhealing (N = 20)	p-Value
Gender (M/F)	15/5	16/4	0.708
Age (years)	59.2 ± 10.17	59.75 ± 8.42	0.787
HbA1C (mg%)	9.72 ± 2.9	10.77 ± 2.7	0.273
MMP-9 tissue (ng/ml)	1,186.62 ± 272.08	2,555.06 ± 993.78	0

**Figure 1:**
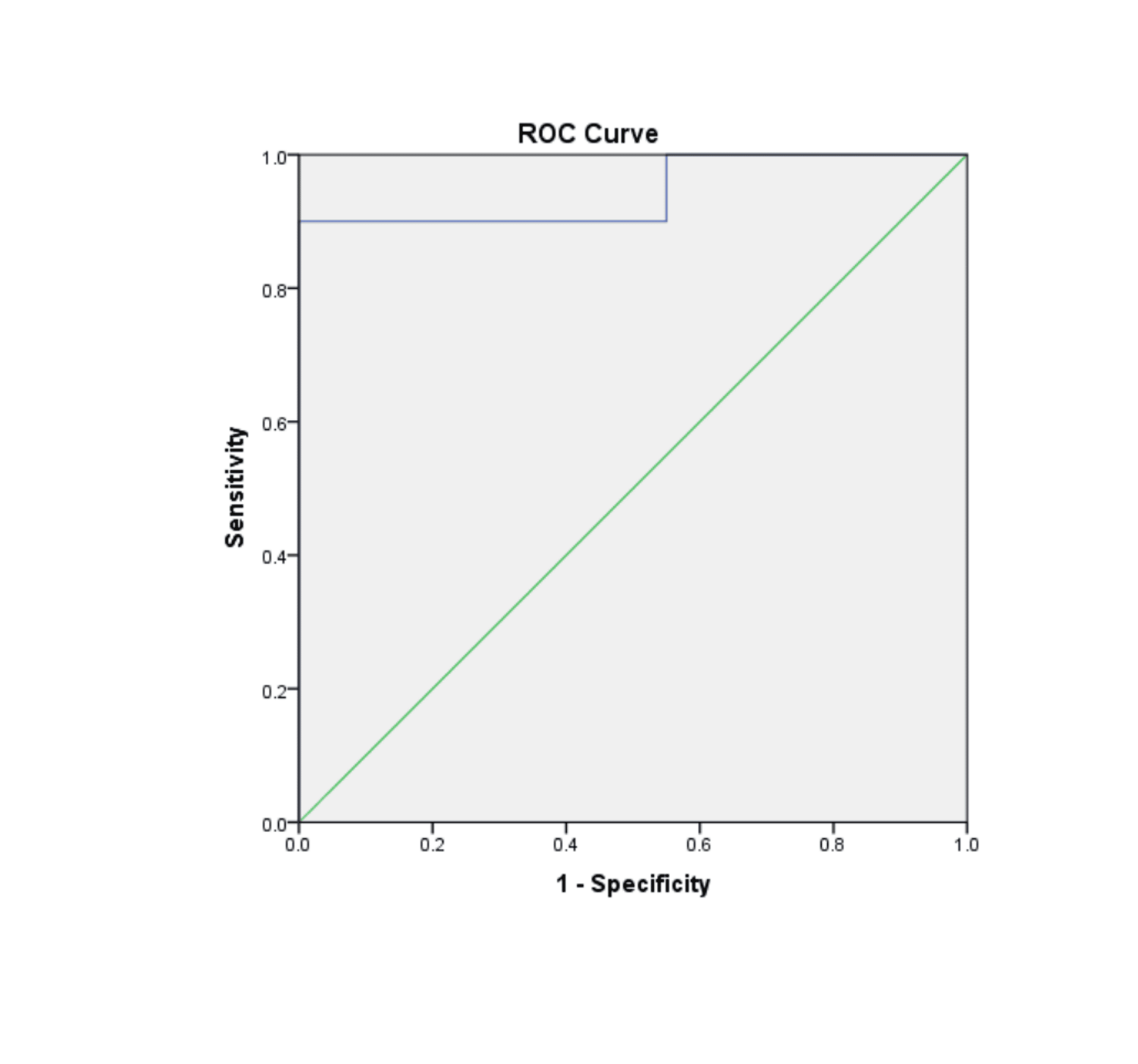
ROC curve showing the predictive ability of MMP-9 in differentiating between healing and nonhealing groups The AUC is 0.945, indicating excellent discriminatory power. AUC: area under the curve; MMP-9: matrix metalloproteinase-9; ROC: receiver operating characteristic

We performed receiver operating characteristic (ROC) analysis to assess the predictive value of MMP-9 in distinguishing between the healing and nonhealing groups. The area under the curve (AUC) typically ranges from 0.5 to 1, with values closer to 1 indicating higher discriminatory ability. As shown in Figure 1, the ROC curve demonstrated that MMP-9 measurement was the best predictor of wound healing (AUC = 0.945; P < 0.01).

The sensitivity and specificity were 90% and 100%, respectively, with outcomes predicted correctly in 94% of cases (Table 2).

**Table 2:** AUC for tissue MMP-9 concentration AUC: area under the curve; MMP-9: matrix metalloproteinase-9

Area	SE	p-Value	Asymptotic 95% CI	
			Lower bound	Upper bound
0.945	0.039	0	0.868	1

**Figure 2:**
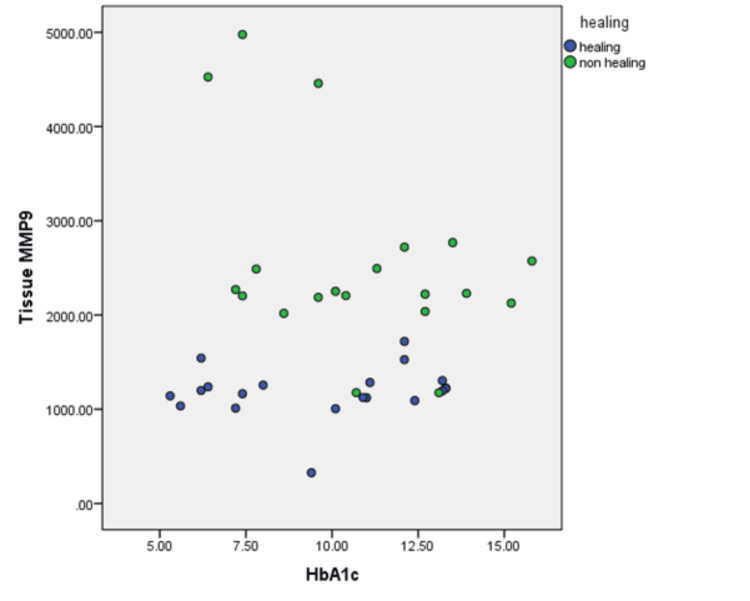
Correlation between tissue MMP-9 concentration and HbA1c levels in subjects with wound healing n = 40, Spearman’s rank correlation: rs = 0.146, p = 0.37 HbA1c, hemoglobin A1C; MMP-9: matrix metalloproteinase-9

A Spearman’s rank-order correlation was conducted to assess the relationship between tissue healing, nonhealing status, and glycated hemoglobin. In the correlation between tissue MMP-9 concentration and HbA1c levels in subjects with wound healing, a weak positive correlation was observed, but it was not statistically significant (Spearman’s rank correlation: rs(38) = 0.146, p = 0.37) (Figure 2).

There was a weak positive correlation between MMP-9 concentration in healing tissue and glycated hemoglobin, but it was not statistically significant (rs(18) = 0.247, p = 0.294). Similarly, in the correlation between MMP-9 concentration in nonhealing tissue and glycated hemoglobin, a weak negative correlation was observed, which was also not statistically significant (rs(18) = -0.239, p = 0.309) (Table 3).

**Table 3:** Correlation between MMP-9 concentration in wound tissue and Gly Hb Gly Hb: glycated hemoglobin; MMP-9: matrix metalloproteinase-9

Groups	Gly Hb r value	p-Value
Healing MMP-9 (ng/ml)	0.247	0.294
Nonhealing MMP-9 (ng/ml)	-0.239	0.309

## Discussion

Our study found that unhealed diabetic wound exudates had higher MMP-9 concentrations than healed wound exudates. By carefully selecting diabetic patients without arteriopathy, infection, or medication use that could affect MMP levels, we minimized potential factors that could interfere with MMP measurements. Notably, we identified a cutoff level for MMP-9 of 1,868.57 pg/µg of total protein using ROC analysis. With 90% sensitivity, 100% specificity, and an area under the ROC curve of 0.945, this cutoff level may predict wound healing outcomes.

Under normal conditions, a small amount of MMP-9 is released during the inflammatory phase of healing, allowing the process to progress to the proliferation phase [14]. The high MMP-9 levels in the poor healer group support the idea that excessive MMP-9 may impede healing.

Increased glycation, breakdown of elastin (EL) and collagen (COL), and deposition in the arterial walls are key contributors to vascular problems in DM [17]. MMPs are integral to this process, as they hydrolyze protein components of the vascular ECM. Gelatinases, a subtype of MMPs, can degrade various substrates, including COL, denatured COL (gelatin), EL, laminin, and fibronectin [18]. Gelatinase B (MMP-9) is one such enzyme. Dysregulation of gelatinase activity, associated with vascular remodeling, fibrosis, and inflammation, may contribute to the pathogenesis of diabetic complications [19].

Elevated MMP-9 levels suggest that the inflammatory phase in DFUs is prolonged, leading to inadequate healing. In patients with diabetes, either specific inhibition [10] or deletion [11] of MMP-9 has been shown to accelerate wound healing. Our findings align with this, as MMP-9 was found in significantly higher levels in grade 3 and 4 ulcers compared to grade 1 and 2 ulcers. Similar results have been observed by other researchers [20].

HbA1c, a key marker for evaluating glycemic control, is strongly associated with diabetic complications [21-23]. Deviations from the target HbA1c level increase the risk of poor outcomes. However, in our study, no significant correlation was found between MMP-9 and HbA1c (p > 0.05).

The collection method we used allowed for the direct measurement of MMP local expression in the wound, providing a more accurate representation of the wound’s microenvironment. Our study results were consistent with previous studies measuring MMP levels in wounds and using the same units (pg/μg of protein). Commercially available kits or other quantitative methods that specifically detect the active form of MMP-9 are needed to more accurately relate MMP-9 activity to wound healing outcomes.

Limitations

Several factors could have influenced the outcomes of this study. First, the relatively small patient population limited the statistical power of our results. Second, the high prevalence of medical calcinosis in diabetic arteries contributed to the variability in DFUs, despite the strict inclusion criteria. However, these strict criteria may not fully reflect real-world clinical scenarios, which could limit the generalizability of our findings. Third, predicting wound healing outcomes is challenging, as the location of the ulcer may impact the prognosis. Fourth, we did not assess the levels of tissue inhibitors of MMPs (TIMPs), which play a role in promoting wound healing. The MMP/TIMP ratio has been shown to affect wound healing [13].

## Conclusions

The development of MMP inhibitors for the treatment of DFUs is still ongoing, but it is essential to find the right balance between promoting healing and preventing excessive ECM remodeling. A comprehensive approach that considers all these factors is necessary for effective DFU treatment. Additional factors, such as immune system dysfunction and diabetes-induced microvascular complications, also contribute to the healing process of DFUs.

MMP-9 expression serves as a marker of poor wound healing. MMP-9 levels in the wound at the time of presentation may help predict the healing trajectory and guide the development of personalized treatment plans for each DFU patient.
